# Catalytic Interactions and Molecular Docking of Bile Salt Hydrolase (BSH) from *L. plantarum* RYPR1 and Its Prebiotic Utilization

**DOI:** 10.3389/fmicb.2016.02116

**Published:** 2017-01-05

**Authors:** Ruby Yadav, Puneet K. Singh, Anil K. Puniya, Pratyoosh Shukla

**Affiliations:** ^1^Enzyme Technology and Protein Bioinformatics Laboratory, Department of Microbiology, Maharshi Dayanand UniversityRohtak, India; ^2^Division of Dairy Microbiology, Indian Council of Agricultural Research (ICAR) – National Dairy Research Institute (NDRI)Karnal, India; ^3^College of Dairy Science and Technology, Guru Angad Dev Veterinary and Animal Sciences UniversityLudhiana, India

**Keywords:** probiotics, prebiotic utilization, molecular docking, Glycocholic acid, bile salt hydrolase (BSH), *Lactobacillus plantarum*

## Abstract

Prebiotics are the non-digestible carbohydrate, which passes through the small intestine into unmetabolized form, reaches the large intestine and undergoes fermentation by the colonic bacteria thus; prebiotics stimulate the growth of probiotic bacteria. Further, bile salt hydrolase (BSH) is an enzyme that catalyses the deconjugation of bile salt, so it has enormous potential toward utilizing such capability of *Lactobacillus plantarum* RYPR1 toward detoxifying through BSH enzyme activity. In the present study, six isolates of *Lactobacillus* were evaluated for the co-aggregation assay and the isolate *Lactobacillus plantarum* RYPR1 was further selected for studies of prebiotic utilization, catalytic interactions and molecular docking. The prebiotic utilization ability was assessed by using commercially available prebiotics lactulose, inulin, xylitol, raffinose, and oligofructose P95. The results obtained revealed that RYPR1 is able to utilize these probiotics, maximum with lactulose by showing an increase in viable cell count (7.33 ± 0.02 to 8.18 ± 0.08). In addition, the molecular docking of BSH from *Lactobacillus plantarum* RYPR1 was performed which revealed the binding energy –4.42 and 7.03 KJ/mol. This proves a considerably good interactions among BSH and its substrates like Taurocholic acid (–4.42 KJ/mol) and Glycocholic acid (–7.03 KJ/mol). These results from this study establishes that *Lactobacillus plantarum* RYPR1 possesses good probiotic effects so it could be used for such applications. Further, molecular dynamics simulations were used to analyze the dynamic stability of the of modeled protein to stabilize it for further protein ligand docking and it was observed that residues Asn12, Ile8, and Leu6 were interacting among BSH and its substrates, i.e., Taurocholic acid and Lys88 and Asp126 were interacting with Glycocholic acid. These residues were interacting when the docking was carried out with stabilized BSH protein structure, thus, these residues may have a vital role in stabilizing the binding of the ligands with the protein.

## Introduction

Probiotics are live microbial food supplements which, when administrated in adequate amounts, exerts various health benefits to consumers (Vinderola et al., 2008). Probiotics is a promising field in dairy and food industry with tremendous growth potential (Mitropoulou et al., 2013). These bacteria exert various health benefits to the host, such as immunomodulation, lipid and cholesterol reduction, anticancer, antimicrobial, antiallergic, antioxidative properties, prevention of gastrointestinal infections, improvement of lactose metabolism, etc. (Lee et al., 2014). Probiotics produce diverse inhibitory substances (organic acids, antimicrobial substances, exoploysaccharides, bacteriocins etc.) which depress growth of pathogenic microorganisms in the gut (Pessione, 2012; Maldonado et al., 2015; Yadav and Shukla, 2015). There are various *in vitro* tests for the selection and study of functional properties of a probiotic strain. The co-aggregation study of probiotic bacteria with pathogens helps in evaluating the pathogen interaction with bacteria which prevents pathogen colonization in the gut (Gupta and Malik, 2007). The interactions of probiotics with prebiotics have a beneficial role in improving the growth of normal microflora, resulting in immune system modulation of the host. A number of systems biology tools have been studied to comprehend the interactions between microorganisms and plant or human cell (Kumar et al., 2016). Furthermore, various genetic modifications which involve the introduction of desired genes may also have a constructive impact in the probiotic field (Gupta and Shukla, 2015). There are many reports on molecular docking of enzymes, which gives good insights of various protein interactions and their effective binding patterns (Singh and Shukla, 2011, 2014; Singh et al., 2011, 2016; Karthik et al., 2012; Baweja et al., 2015, 2016).

In the present study probiotic properties, prebiotic utilization and the molecular docking of *Lactobacillus plantarum* RYPR1, isolated from indigenous fermented beverage raabadi was performed. The development of nano-encapsulated probiotics is an emerging field and showing new possibilities of probiotics in food industry. The viability of probiotic bacteria in the human body could extend by using nanoencapsulated bacteria, so that it could show better interaction with receptors of the gastrointestinal tract. The results reported from our previous studies showed that *L. plantarum* RYPR1 possess good antimicrobial activity so its probiotic effect could be further improved by the development of nano-encapsulated probiotics by using nanotechnology applications.

## Materials and Methods

### Isolation and Probiotic Properties of *Lactobacillus* Isolates

A total of 11 curd (6) and raabadi (5) samples were collected from different regions of Haryana, India following the standard microbiological protocols. Moreover, the Kanji (fermented beverage made up of carrot) samples were prepared in laboratory under aseptic conditions for isolating lactic acid bacteria. The isolation and purification of lactic acid bacteria, was done using De Man Rogose Sharpe (MRS) medium (Goyal et al., 2013). The purified cultures isolated from these samples were tested for grams staining, endospore staining, catalase test and further tested for various probiotic properties as reported in our previous studies (Yadav et al., 2016).

### Co-aggregation Assay

Co-aggregation involves the process of aggregation of bacterial cells of more than one type (Kumar et al., 2012). Co-aggregation ability provides a close interaction of probiotic bacteria with pathogenic bacteria (Singh et al., 2012). In this experiment, *E. coli* was taken as indicator organism which can co-aggregate with selected isolates. Overnight grown Lactobacilli (16–18 h) and *E. coli* cultures were centrifuged (10000 rpm, 15 min) and the pellets obtained were washed twice with phosphate buffer saline (PBS) solution (pH 6.0). The pellets were resuspended in PBS, vortexed and the absorbance was set 0.5 at 600 nm. After this, 500 μl of culture and 500 μl of the pathogen were mixed and optical density (OD) was measured at 600 nm and incubated at 37°C for 2 h. Upper phase was carefully removed and absorbance was measured at 600 nm. Decrease in absorbance was taken as a measure of cell co-aggregation. The co-aggregation percentage was calculated by using the following formula:

Percent co-aggregation-[(OD1+OD2)−2(OD3)/(OD1+OD2) × 100]

OD_1_: optical density of *Lactobacillus* isolates, OD_2_: optical density of *E. coli*, OD_3_: optical density of mixture.

### Prebiotic Utilization

Commercially available five prebiotics Lactulose, Xylitol, D+ raffinose, Inulin and Oligofructose P95 were used for the test. Prebiotics were solubilized in distilled water and filter sterilized. Isolates were inoculated with 3 ml of modified MRS medium (2% of each probiotic) and incubated at 37°C for 24 h under anaerobic conditions. OD of each culture was measured at 560 nm and the cell growth rate was calculated by using the formula:

Prebiotic utilization-(MRSp-MRSb) × 100/MRSg-MRSb

### Molecular Dynamic Simulation

Molecular dynamics was performed with Gromacs 4.5.5 using the Gromos96 force field. All the water molecules were deleted and polar hydrogen atoms were added. The hydrogen atoms were minimized with 500 steps of Steepest Descent (SD) optimization; spc water was added in a sphere with a radius of 18 Å around the reaction center (the Cl and NA ions). Before the unconstrained MD simulation, the solvent was subjected to 1000 steps of SD minimization, and equilibrated for 2.5 ps at 300 K with solute fixed. The production simulation was carried out for 5,000 ps (5 ns). The average conformation was calculated for the desired represented frame of MD simulations. This was achieved by averaging the snapshots of the last 500 ps, then choosing a typical structure with the lowest RMSD to the average conformation, and using this in the binding mode analysis.

### Molecular Docking and Analysis of Bile Salt Hydrolase (BSH)

The Bile Salt Hydrolase (BSH) activity of the selected isolate as reported previously was further taken as standard for the catalytic interaction. The study of enzyme modeling was performed with SWISS-MODEL, it is a fully automated protein structure homology-modeling server, accessible via the ExPASy web server, or from the program DeepView (Swiss Pdb-Viewer). SWISS-MODEL provides graphical representation as well as numerical calculations for the alignment of structures. In SWISS-MODEL we have to submit sequence of protein of which we have to model structure and it will provide a structure after few hours (Arnold et al., 2006; Guex et al., 2009; Kiefer et al., 2009). The Stereochemical quality of a protein was checked by PROCHECK it analyses the structure by analyzing residue-by-residue geometry and overall structural geometry of the modeled structure (Laskowski et al., 1993, 1996). ERRAT Analyzes the statistics of non-bonded interactions between different atom types and compare with the highly refined structures.

## Results

### Isolation and Probiotic Properties of *Lactobacillus* Isolates

A total of 119 isolates were isolated from curd, kanji, and raabadi samples and 90 were purified. On the basis of colony morphology, gram staining, endospore staining and catalase test 54 isolates were identified and selected as *Lactobacillus* isolates. These isolates were further tested for probiotic properties. It has been shown that among the tested *Lactobacillus* isolates, isolate *Lactobacillus plantarum* RYPR1 as identified by 16S rRNA sequencing and phylogenetic analysis (GenBank accession number KX620369) showed the maximum probiotic potential and it was selected for further studies. Moreover, cell co-aggregation, prebiotic utilization, BSH activity and *in silico* studies are presented in this paper.

### Cell Co-aggregation

**FIGURE 1:**
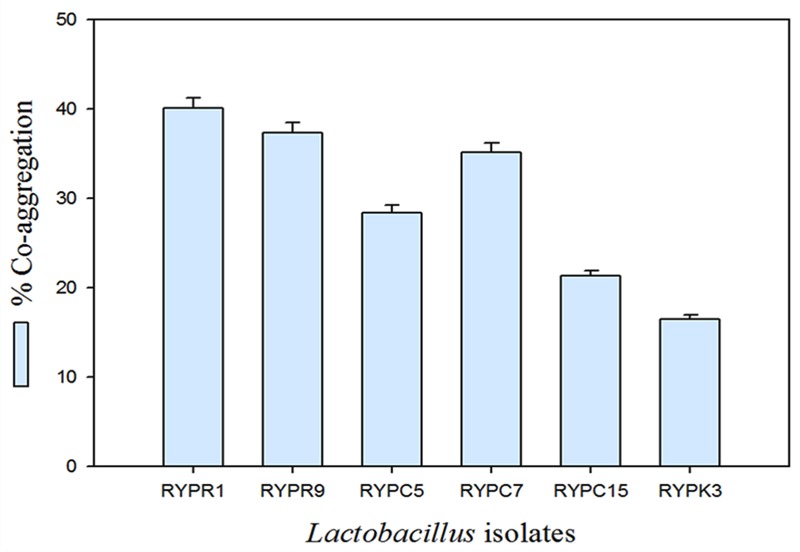
**Co-aggregation percent of selected isolates**.

Cell co-aggregation involves interaction of probiotic microorganism with surface components of pathogenic bacteria. The co-aggregation activity involves biofilm formation which helps the host by prevention of pathogen colonization in the gut. The co-aggregating cell clumps together and settled at the bottom of the tube, resulting in decreasing absorbance of suspension. Percent co-aggregation of selected isolates ranged from 17 to 40% (**Figure 1**). Strain RYPR1 showed highest co-aggregation potential followed by RYPR9.

### Prebiotic Utilization

The viable cell counts of *L. plantarum* with prebiotics after 24 h incubation are presented in **Table 1**. Based on viable cell count, it was observed that isolate RYPR1 showed the maximum survival with lactulose followed by raffinose and inulin. So, with this study it was concluded that RYPR1 growth could be stimulated by tested probiotics.

**Table 1:** Prebiotic substrate utilization by *Lactobacillus plantarum* RYPR1 after 24 h incubation.

Prebiotic substrate	log cfu/ml (after incubation)	
	0 h	24 h
Lactulose	7.33 ± 0.02	8.18 ± 0.08
Xylitol	7.1 ± 0.01	6.44 ± 0.07
D+ raffinose	7.98 ± 0.17	8.05 ± 0.10
Inulin	7.91 ± 0.09	7.90 ± 0.04
Oligofructose P95	7.62 ± 0.10	6.88 ± 0.09

### Molecular Docking Analysis of BSH

Isolate RYPRI was grown in the presence of bile salts (sodium tauroglycocholate, sodium taurocholate, sodium taurodeoxycholate) to evaluate its ability to hydrolyze high concentration of bile salts. The results obtained from this study concluded that RYPR1 is not only able to survive the toxicity of bile salts, but also carries out bile salt deconjugation which helps in the colonization of bacteria to intestinal epithelial cells. The results from *in vitro* studies were further confirmed by *in silico* studies.

#### Homology Modeling and Structure Validation

The sequence for modeling was submitted to Swiss Model^1^ for structural modeling. During the study, the template chosen for modeling demonstrated similarity of 70.01% with 4wl3 chain B, Crystal structure determination of BSH from *Enterococcus feacalis* having resolution of 2.01 Å. Structure validation was performed using SAVES server. ERRAT Overall quality factor was obtained was 96.349 which represent a stable structure. PROCHECK also exhibited favorable result for protein model to proceed for molecular docking.

#### Molecular Dynamic Simulation and Docking Analysis

**FIGURE 2:**
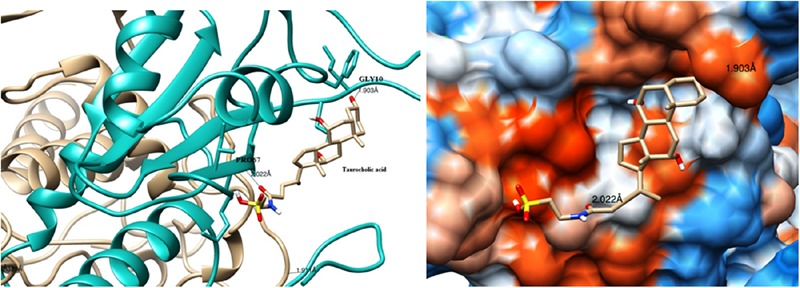
**Substrate binding studies on the surface of Bile Salt Hydrolase (BSH) from *L. plantarum* with Taurocholic acid.** Gly10, Pro67 involved in the interaction with 1.903 and 2.022 Å; of h-bond, respectively, with the enzyme.

**FIGURE 3:**
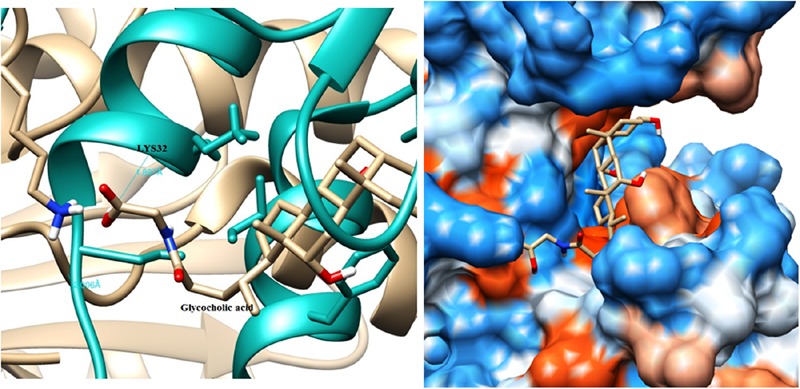
**Substrate binding studies on the surface of BSH from *L. plantarum* with Glycocholic acid.** Lys32 formed hydrogen bonding with the Glycocholic acid with bond length of 1.879 Å.

**FIGURE 4:**
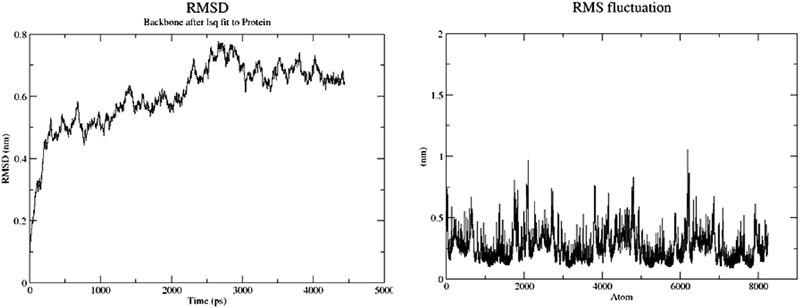
**Root mean square deviation (RMSD) and root mean square fluctuation (RMSF) of BSH**.

**FIGURE 5:**
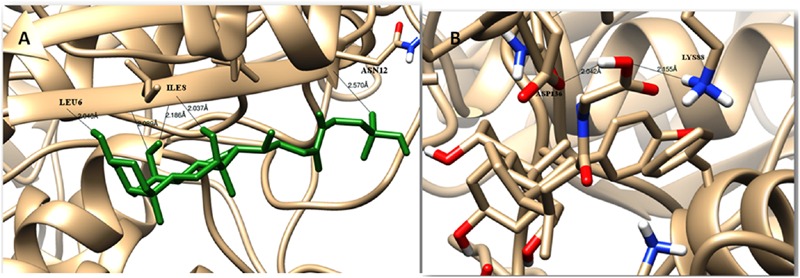
**Substrate binding studies on the surface of BSH from *L. plantarum* with Taurocholic acid (A)** and Glycocholic acid **(B)** after stabilizing the protein.

The modeled BSH from *L. plantarum* was stabilized by molecular dynamic simulation of 5,000 ps (5 ns) (**Figure 4**). Further, the stabilized protein was docked with Taurocholic acid and Glycocholic acid and the result was compared with the docking result of unstabilized protein. The BSH activity of the selected isolate as reported previously was further taken as standard for the catalytic interaction of BSH with Taurocholic acid and Glycocholic acid. The docking was carried out with AutoDock4^2^. In AutoDock4, enzyme BSH from *L. plantarum* docked with Taurocholic acid and Glycocholic acid The result was recorded as the least binding energy with Glycocholic acid as –7.03 KJ/mol, followed by docking with Taurocholic acid with –4.42 KJ/mol, least binding energy signifies the strong binding between substrate and enzyme. The minimum inhibition constant of Glycocholic acid also came to be minimum, i.e., 252.24 μl. Lys32 formed hydrogen bonding with the Glycocholic acid with bond length of 1.879 Å. Gly10, Pro67 involved in the interaction with 1.903 and 2.022 Å of h-bond, respectively, with the enzyme (**Figures 2** and **3**). The comparison of the minimum binding energy of both the substrate observed with AutoDock **Table 2**. The docking result of BSH from *L. plantarum* docked with Taurocholic acid and Glycocholic acid after stabilizing the protein gave different results. The least binding energy raised, however, Lys88 was involved in the interaction with the Glycocholic acid, which implies that Lys plays an important role in the active site of BSH (**Figure 5**).

**Table 2:** Docking studies of Bile Salt Hydrolase (BSH) from *L. plantarum* with Taurocholic acid and Glycocholic acid.

Ligand	Protein	Binding energy (kc/mol)	Inhibition constant	Hydrogen bonds	Hydrogen bond length
Taurocholic acid	BSH	–4.42	580.35 mM	Gly10, Pro67	1.903 and 2.022 Å
Taurocholic acid (after stabilizing protein)	BSH (after stabilizing protein)	–3.01	6.26 mM	Asn12, Ile8 and Leu6	2.570, 2.037, and 2.940 Å
Glycocholic acid	BSH	–4.91	252.24 mM	Lys32	1.879 Å
Glycocholic acid (after stabilizing protein)	BSH (after stabilizing protein)	–3.45	7.66 mM	Asp136 and Lys88	2.042 and 2.155 Å

## Discussion

The objective of the present study was to assess the probiotic potential of *Lactobacillus* isolates from food samples. Among tested isolates, isolate *L. plantarum* RYPR1 showed good probiotic potential and therefore it was selected for further studies. It is a commonly used and well studied probiotic strain and it is used for development of various probiotic based food products (Pisano et al., 2008). *In vitro* assessment of co-aggregation ability of isolate with *E. coli* was also studied as it is also an important selection criterion. The co-aggregation rate was observed for 2 h and it was observed that RYPR1 showed the maximum co-aggregation (40%). Similar studies were conducted by Ramos et al. (2013) and reported that *L. plantarum* CH41 showed highest co-aggregation ability with *E. coli.* Furthermore, another study reported that *L. plantarum* S1 showed the maximum co-aggregation ability (37–41%) with common enteric pathogens (Jankovic et al., 2012). Based on viable cell count after 24 h incubation with probiotics it was analyzed that RYPR1 is able to utilize probiotics. The prebiotic study of RYPR1 with commercially available prebiotics is important as prebiotics stimulates their growth in GIT (Macfarlane et al., 2007). A few studies have been reported with *L. plantarum* which confirms a correlation of prebiotics and β-galactosidase enzyme (Pennacchia et al., 2006). Moreover, few other researchers conducted studies related to probiotics functionality, safety, γ-amino butyric acid production; genomics and metabolomics etc., however, the catalytic binding and interaction studies are included in the present work which provides further lead to carry out such work in prebiotic utilizations. (Devi et al., 2016; Shekh et al., 2016; Stefanovic et al., 2017). In the present study, the BSH activity of RYPR1 was further taken as standard for studying the catalytic interaction of BSH with Taurocholic acid and Glycocholic acid. The study was done using SWISS-MODEL which provides a model structure of tested protein. The BSH enzyme from *L. plantarum* was docked with Taurocholic acid and Glycocholic acid and the results revealed that Glycocholic acid showed the least binding energy (–7.03 KJ/mol) followed by Taurocholic acid (–4.42 KJ/mol). Minimum the binding energy more will be the interaction resulting in good BSH activity. The study showed that *L. plantarum* RYPR1 is able to hydrolyse these salts, which assume that it can survive the toxicity of bile salts and also carry out deconjugation of these salts which may help in their colonization in the intestine.

## Conclusion

In our previous study, we have reported the probiotic potential of *L. plantarum* RYPR1. Consequently, concluded that it can be used as a starter culture for the preparation of probiotic food products. The results of co-aggregation studies with pathogenic bacteria indicate that *L. plantarum* RYPR1 could be used to prevent pathogen colonization in the gut. The present study concludes that *L. plantarum* RYPR1 is able to utilize most of the prebiotics. However, the best growth was observed among lactulose followed by raffinose. Thus, we could use these prebiotics along with our selected strain to develop an effective synbiotic, which can stimulate the overall human gut microflora. Due to its good antimicrobial activity and other aspects, this indigenous isolate could be used in other relevant applications. Furthermore, the catalytic interaction of BSH with Taurocholic acid and Glycocholic acid proves further that it can act as an excellent source for various probiotic applications.

## Author Contributions

All authors listed, have made substantial, direct and intellectual contribution to the work, and approved it for publication.

## Conflict of Interest Statement

The authors declare that the research was conducted in the absence of any commercial or financial relationships that could be construed as a potential conflict of interest.

## Footnotes

1 http://swissmodel.expasy.org

2 http://autodock.scripps.edu
